# Supplementary material for: Transcriptomic Insights into Mechanisms of Early Seed Maturation in the Garden Pea (Pisum sativum L.)
Source: Cells. 2020 Mar 23;9(3):779. doi: 10.3390/cells9030779 (PMC7140803; doi:10.3390/cells9030779)

Sprint−2(20) vs Zhongv

Global Summary

%DE = 0.84

genes with fdr < 0.2 = 1645 (820 + / 825 −)

genes with fdr < 0.1 = 1556 (767 + / 789 −)

genes with fdr < 0.05 = 1334 (662 + / 672 −)

genes with fdr < 0.01 = 1041 (503 + / 538 −)

genes in genesets = 0

<FC> = 0

<t−score> = −2.75

<p−value> = 0

<fdr> = 0.16

Portrait

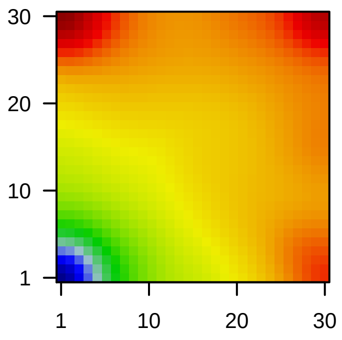

Top 100 DE genes

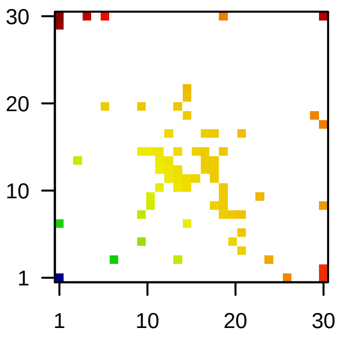

Global Genelist

Rank	ID	log(FC)	p−value	fdr	Metagene	Description
1	TRINITY_DN1	−0.01	1e−09	0.00000018	x 12	
2	TRINITY_DN1	−0.01	1e−09	0.00000017	x 13	
3	TRINITY_DN3	−0.01	2e−09	0.00000017	x 15	
4	TRINITY_DN4	0	2e−09	0.00000018	x 14	
5	TRINITY_DN5	−0.01	2e−09	0.00000017	x 14	
6	TRINITY_DN3	−0.01	2e−09	0.00000021	x 17	
7	TRINITY_DN1	−0.01	3e−09	0.00000017	x 14	
8	TRINITY_DN4	−0.01	3e−09	0.00000017	x 14	
9	TRINITY_DN1	−0.03	4e−09	0.00000015	x 11	
10	TRINITY_DN3	0	4e−09	0.00000017	x 15	
11	TRINITY_DN3	0	4e−09	0.00000019	x 10	
12	TRINITY_DN2	−0.04	5e−09	0.00000014	x 20	
13	TRINITY_DN4	−0.01	5e−09	0.00000017	x 14	
14	TRINITY_DN6	−0.08	5e−09	0.00000012	x 11	
15	TRINITY_DN1	0	6e−09	0.00000017	x 14	
16	TRINITY_DN4	−0.01	6e−09	0.00000015	x 21	
17	TRINITY_DN9	0	7e−09	0.00000017	x 14	
18	TRINITY_DN3	−0.04	7e−09	0.00000013	x 12	
19	TRINITY_DN2	0	7e−09	0.00000017	x 15	
20	TRINITY_DN1	0	7e−09	0.00000017	x 15	

p−values

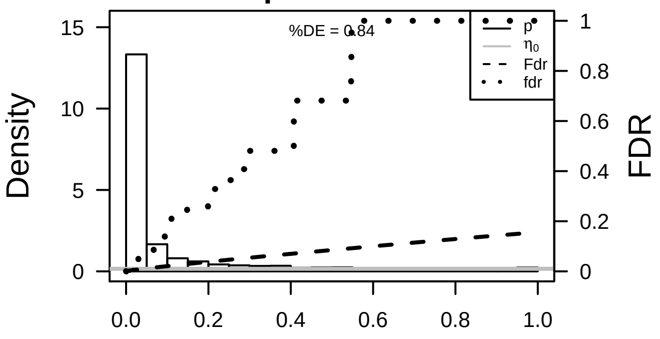

Supplement: Supplementary file 1 [file cells-09-00779-s001.zip › Supplementary materials/FileS4.zip!/FileS3/TFs - Results/Summary Sheets - Differences/Reports/Sprint.2.20..vs.Zhongwan.6.10..pdf]
